# Evaluation of Antigen Productivity and Inactivation Kinetics of a Recombinant Foot-and-Mouth Disease SAT1 Vaccine Strain

**DOI:** 10.3390/v18050537

**Published:** 2026-05-06

**Authors:** Jae Young Kim, Sun Young Park, Gyeongmin Lee, Seung-A Hwangbo, Giyoun Cho, Jong-Hyeon Park, Young-Joon Ko

**Affiliations:** Center for Foot-and-Mouth Disease Vaccine Research, Animal and Plant Quarantine Agency, 177 Hyeoksin 8-ro, Gimcheon-si 39660, Gyeongsangbuk-do, Republic of Korea; ivorikim@korea.kr (J.Y.K.); sun3730@korea.kr (S.Y.P.); lgm6004@korea.kr (G.L.); hbsa230@korea.kr (S.-A.H.); libretto@korea.kr (G.C.); parkjhvet@korea.kr (J.-H.P.)

**Keywords:** foot-and-mouth disease, SAT1 BOT-R, vaccine, antigen productivity, inactivation kinetics

## Abstract

The Republic of Korea has implemented routine vaccination against foot-and-mouth disease virus (FMDV) in livestock using a bivalent vaccine comprising serotypes O and A following the massive FMD outbreak in 2010, while antigens for the remaining serotypes are maintained in overseas antigen banks. The recent geographic expansion of FMDV Southern African Territories 1 (SAT1) beyond Africa underscores the need for enhanced preparedness in previously unaffected regions. In this study, we evaluated the SAT1 BOT-R strain as a candidate vaccine seed for potential domestic vaccine production by optimizing antigen production conditions, assessing scalability, determining virus inactivation parameters, and examining immunogenicity in pigs. Optimal antigen yield was achieved at 20 h−24 h post infection with a multiplicity of infection of 0.005−0.01, with production remaining stable under mildly alkaline conditions. Antigen productivity was consistently maintained during scale-up from shake flasks to a bioreactor, yielding up to 9.5 μg/mL. Complete virus inactivation was achieved using binary ethylenimine at 2 mM for 24 h at 26 °C. Vaccines formulated from both flask- and bioreactor-derived antigens elicited comparable neutralizing antibody responses in pigs, reaching a median titer of 1:500 following booster immunization. Collectively, these findings demonstrate that the SAT1 BOT-R strain is a viable and scalable candidate for SAT1 antigen banking and future domestic vaccine production, providing a practical framework for strengthening national preparedness against potential incursions of FMDV SAT1.

## 1. Introduction

Foot-and-mouth disease (FMD) is a highly contagious viral disease affecting cloven-hoofed animals, including cattle, pigs, and goats, and is characterized by vesicular lesions on the teats, oral cavity, and feet. In the Republic of Korea, FMD is classified as a Class I notifiable animal disease. Outbreaks cause not only substantial direct economic losses due to reduced productivity and culling, but also severe restrictions on international trade in animals and animal products [1,2].

FMD virus (FMDV), a member of the family *Picornaviridae* and genus *Aphthovirus*, is a non-enveloped virus containing a single-stranded RNA genome. Its capsid is composed of four structural proteins, VP1, VP2, VP3, and VP4, whereas the viral genome also encodes eight non-structural proteins involved in replication and virus processing [3,4]. Based on cross-protection characteristics, FMDV is classified into seven serotypes: O, A, Asia 1, C, Southern African Territories (SAT) 1, SAT2, and SAT3. Among the structural proteins, VP1 has been recognized as the major determinant of antigenic variation and serotype discrimination [4]. During virion assembly, VP0, VP3, and VP1 first form a protomer, and five protomers subsequently assemble into a pentamer. Twelve pentamers then associate to generate the viral precursor particle. Upon encapsidation of viral RNA, VP0 cleaves into VP2 and VP4, leading to the mature 146S virion [5,6,7].

Although FMDV is composed of four structural proteins, the most relevant vaccine antigen is not an individual capsid protein but the intact 146S particle itself. FMDV particles are readily dissociated into pentamers under acidic conditions and by heat treatment. Even though pentamers and intact 146S particles consist of the same structural proteins, their immunogenicities differ substantially because of their distinct conformational properties. This difference results in reduced neutralizing antibody responses and, consequently, inferior protective efficacy in pentamer-vaccinated animals [8,9,10]. Therefore, maximizing the yield of intact 146S antigen is a critical objective in FMD vaccine manufacturing.

Because FMDV spreads extremely rapidly via direct contact and air, immediate control of the outbreak source is essential once FMDV infection occurs. The most effective preventive strategy is vaccination before exposure [11,12,13]. Since the nationwide large-scale FMD outbreaks in 2010–2011, the Republic of Korea has implemented routine FMD vaccination programs for cattle, pigs, and goats. A bivalent vaccine containing serotypes O and A, which have previously caused outbreaks in Republic of Korea, is currently used in the field. In contrast, antigens for the remaining five serotypes (C, Asia1, SAT1, SAT2, and SAT3), which have not been reported domestically, are maintained in overseas antigen banks at foreign FMD vaccine manufacturing facilities.

To localize FMD vaccine production, a domestic FMD vaccine manufacturing facility is planned to be established in Republic of Korea, and the overseas antigen bank is expected to be replaced with a national antigen bank in the future. In preparation for this transition, our institute has been developing vaccine strains representing multiple FMDV serotypes. Among these candidates, SAT1 BOT-R has previously been shown by another research group in our institute to confer protective efficacy against viral challenge in pigs [14]. The present study aimed to establish culture conditions capable of supporting adequate antigen production from the SAT1 BOT-R strain, to assess whether antigen yields could be reproducibly maintained during scale-up, and to determine whether the virus could be effectively inactivated in compliance with international standards for potential commercial applications, including use in an antigen bank.

## 2. Materials and Methods

### 2.1. Cells and Virus

BHK-21 (C-13; ATCC CCL-10, Manassas, VA, USA) and LFBK cells obtained from the Plum Island Animal Disease Center were propagated under standard conditions. Both cell lines were maintained in Dulbecco’s modified Eagle’s medium (DMEM; Corning (Corning Inc., Corning, NY, USA)) supplemented with 10% fetal bovine serum (Gibco) and 1% antibiotic–antimycotic solution (Gibco, Waltham, MA, USA).

Suspension-adapted baby hamster kidney cells (BHK-21), which were developed by the Animal and Plant Quarantine Agency (APQA) and the Korea Research Institute of Bioscience and Biotechnology in Republic of Korea, were maintained in CD BHK-21 Production medium (Gibco, Waltham, MA, USA) at 110 rpm in a 37 °C, 5% CO_2_ shaking incubator. Cell density and viability were analyzed by trypan blue exclusion assay using an automated cell counter (Vi-Cell XR; Beckman Coulter, Brea, CA, USA). The SAT1 BOT-R virus used in this study was a recombinant strain generated by reverse genetics through replacement of the structural protein-coding region of an FMDV O1 Manisa full-length infectious clone with the corresponding region of SAT1 BOT 1/68 (AY593845) as previously described [14].

### 2.2. Optimization of Culture Conditions for Antigen Production

To determine suitable infection conditions for antigen production, suspension BHK-21 cells were seeded in CD BHK-21 Production medium (Gibco) at 3 × 10^5^ cells/mL and cultured for 3.5 days to reach approximately 3 × 10^6^ cells/mL. The existing culture medium was removed while retaining the cells and replaced entirely with fresh medium. The cells were then infected with SAT1 BOT-R at MOIs ranging from 0.001 to 0.05 and 3 mM CaCl_2_ (Sigma-Aldrich, St. Louis, MO, USA) was simultaneously added at the time of virus inoculation. Cultures were maintained at 37 °C in a 5% CO_2_ atmosphere with shaking at 110 rpm. Samples were collected at designated time points after infection, and cell debris was removed by low-speed centrifugation prior to further analysis. To examine the effect of pH on antigen productivity, BHK-21 suspension cultures were prepared under the same conditions and adjusted to pH values ranging from 6.0 to 9.0 at the time of virus inoculation. Cells were infected at an MOI of 0.005 with SAT 1 BOT-R and incubated for 24 h under the same temperature and atmospheric conditions described above. The culture was clarified by centrifugation at 4000× *g* for 20 min at 4 °C and used for virus titration and 146S antigen quantification.

### 2.3. Virus Titration

Virus infectivity was quantified by endpoint titration in adherent BHK-21 cells. Infectious titers were calculated using the Spearman–Karber method and expressed as 50% tissue culture infectious dose per milliliter (TCID_50_/mL) [15].

### 2.4. Quantification of FMDV Particles

The clarified virus supernatants were mixed with chloroform (Merck KGaA, Darmstadt, Germany) at a 1:1 (*v*/*v*) ratio and vortexed for 5 min. After centrifugation at 4 °C and 3000× *g* for 5 min, the aqueous phase was collected. Benzonase was added at 0.025 units/µL, and samples were incubated at 37 °C for 1 h with agitation. The treated supernatants were then analyzed by size-exclusion HPLC (Agilent Technologies, Santa Clara, CA, USA). The analysis was performed on a TSKgel G4000PWXL (TOSOH Bioscience, Tokyo, Japan; 300 mm × 7.8 mm I.D.) column protected by a PWXL Guardcol (TOSOH Bioscience). Separation was achieved with a mobile phase consisting of 30 mM Tris-HCl and 400 mM NaCl (pH 8.0), delivered at 0.5 mL/min, while detection was performed at 254 nm. Instrument components comprised a quaternary pump with integrated degassing, a temperature-controlled column chamber, an autosampler with cooling capability, and a fraction collector. Chromatographic data were processed using OpenLAB CDS ChemStation (version 3.2.0.620), and antigen concentrations were estimated by integrating peak areas based on previously reported methods [16].

### 2.5. Production of Vaccine Antigen at Different Culture Scales

For flask-scale production, suspension BHK-21 cells were seeded at 3 × 10^5^ cells/mL in working volumes of 40, 200, and 1000 mL using appropriately sized flasks and grown for 3.5 days to reach about 3 × 10^6^ cells/mL. The cells were then infected with SAT1 BOT-R under the optimized infection conditions identified in the preliminary experiments. Virus-containing supernatants were harvested 24 h post infection (hpi) and clarified by centrifugation at 4000× *g* for 20 min at 4 °C. For bioreactor production, suspension BHK-21 cells were grown in a 2 L stirred-tank bioreactor (Sartorius, Göttingen, Germany) equipped with automatic control systems for temperature, dissolved oxygen, and pH. The culture was maintained at 37 °C with dissolved oxygen controlled at 45% air saturation, and agitation was set at 150 rpm. Culture pH was regulated using CO_2_ and sodium hydroxide. Once the target cell density was achieved, the culture temperature was lowered to 4 °C, and agitation, pH control, and air supply were discontinued. The cells were then allowed to settle by gravity for at least 12 h. Subsequently, 90% of the total culture volume was replaced with fresh medium, and the pH was adjusted to the pH 8.0 before infection with SAT1 BOT-R. Supernatants were harvested 24 hpi and clarified by centrifugation at 4000× *g* for 20 min at 4 °C before use in downstream analyses.

### 2.6. FMDV Inactivation Kinetics

Binaryethylenimine (BEI) stock solution (0.1 M) was prepared by dissolving bromoethylamine hydrobromide (Sigma-Aldrich) in 0.2 N sodium hydroxide (Sigma-Aldrich) and incubating the mixture at 37 °C for 1 h. The solution was adjusted to pH 8.5–9.0 and prepared fresh before each experiment. For inactivation kinetics, virus-infected culture supernatants were exposed to BEI at concentrations between 0.5 and 3 mM and incubated at either 26 °C or 37 °C under constant agitation. Sampling was performed periodically, with hourly collections during the initial 6 h followed by a final time point at 24 h to assess remaining infectivity. Immediately after collection, BEI was quenched by adding sodium thiosulfate (Daejung Chemicals & Metals, Siheung, Republic of Korea) to a final concentration of 2% (*v*/*v*).

### 2.7. Animal Experiment

Purified SAT1 BOT-R antigens derived from either flask or bioreactor production were formulated as monovalent experimental vaccines containing 15 µg of inactivated virus per dose. The vaccine formulation consisted of ISA 206 VG adjuvant (Seppic, Paris, France) at a 1:1 (*v*/*v*), supplemented with 1% saponin (Sigma-Aldrich) and 1% aluminum hydroxide gel (General Chemical, Mount Laurel, NJ, USA), and was adjusted to a final dose volume of 2 mL. They were placed in a water bath at 20 °C for 1 h without light exposure and stored at 4 °C. Two-month-old female pigs, weighing 20–25 kg, were seronegative for FMDV. They were maintained under standard farm conditions under veterinary supervision and allocated randomly into three groups: a non-vaccinated control group (*n* = 3), a vaccinated group receiving antigen produced in flask culture (*n* = 5), and a vaccinated group receiving antigen produced in bioreactor culture (*n* = 5), and were immunized intramuscularly twice at a 4-week interval. A minimum of three animals per group is generally required to enable basic statistical analysis; accordingly, the negative control group consisted of three animals. In vaccine studies, unexpected mortality or dropout may occur due to vaccination-related stress and individual variability. Given that the loss of even a single animal in a small experimental group can markedly influence statistical outcomes, the vaccinated group was designed to include five animals to ensure sufficient robustness and allowance for potential attrition. Blood samples were collected weekly post-vaccination (dpv) until one month after the booster immunization. All animal procedures were conducted in accordance with institutional guidelines and were approved by the relevant animal ethics committee of the Animal and Plant Quarantine Agency (APQA; approval no. 2025-1801).

### 2.8. Virus Neutralization Test

Virus neutralization (VN) assays were performed in accordance with the WOAH Terrestrial Manual [17]. Serum samples were heat-inactivated at 56 °C for 30 min, then serially two-fold diluted from 1:4 in 50 µL volumes in 96-well plates. SAT1 BOT-R containing 100 TCID_50_ was added to each well and incubated for 1 h at 37 °C. Subsequently, 50 µL of LFBK cells (0.5 × 10^6^ cells/mL) was added. Plates were incubated at 37 °C with 5% CO_2_ for 2–3 days. Titers are expressed as the final serum dilution in the serum–virus mixture at which 50% of the wells are protected. VN titers were defined as the reciprocal of the highest serum dilution that neutralized 100 TCID_50_ of SAT1 BOT-R and were expressed on a log10 scale.

### 2.9. Statistical Analysis

All experiments were carried out in triplicate, and data are presented as mean ± standard deviation. Statistical analyses were performed using GraphPad Prism Software version 10 (La Jolla, CA, USA). Differences among groups were evaluated by two-way analysis of variance (ANOVA), and values of *p* < 0.05 were considered statistically significant.

## 3. Results

### 3.1. Optimization of Antigen Production Conditions for SAT1 BOT-R

**Figure 1:**
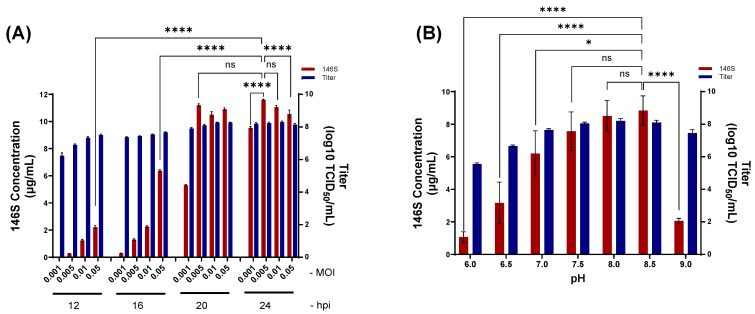
Optimization of SAT1 BOT-R antigen production according to virus-inoculation conditions. (**A**) Virus titers and 146S antigen levels were determined after infection with SAT1 BOT-R at the indicated multiplicity of infection (MOI) and collection at indicated hours post infection (hpi) in the presence of 3 mM CaCl_2_. (**B**) Antigen production was further examined after adjusting the culture medium to different pH values at the time of virus inoculation. Results are shown as mean ± standard deviation. **** *p* < 0.0001; * *p* < 0.05; ns = not significant.

SAT1 BOT-R was inoculated at multiplicities of infection (MOI) of 0.001, 0.005, 0.01, and 0.05 in the presence of 3 mM Ca^2+^, and viruses were harvested at 12, 16, 20, and 24 hpi (Figure 1A). Antigen levels increased over time following infection. At 20 hpi and 24 hpi, no significant differences in antigen production were observed among virus doses above 0.005 MOI. The highest antigen yield was obtained at 0.005 MOI after 24 h of infection, reaching 11.6 μg/mL. Based on these results, the optimal production condition was determined to be infection for 20 h−24 h at an MOI ranging from 0.005 to 0.01.

To further optimize culture conditions, SAT1 BOT-R was inoculated into a 40 mL culture at 0.005 MOI and incubated for 24 h after adjusting the culture medium pH from 6.0 to 9.0 at the time of virus inoculation (Figure 1B). The antigen yields were 1.1 μg/mL at pH 6.0, 3.2 μg/mL at pH 6.5, 6.2 μg/mL at pH 7.0, 7.6 μg/mL at pH 7.5, 8.5 μg/mL at pH 8.0, 8.8 μg/mL at pH 8.5, and 2.1 μg/mL at pH 9.0. No significant differences in antigen yield were observed between pH 7.5 and pH 8.5.

### 3.2. Antigen Productivity of SAT1 BOT-R During Scale-Up

**Figure 2:**
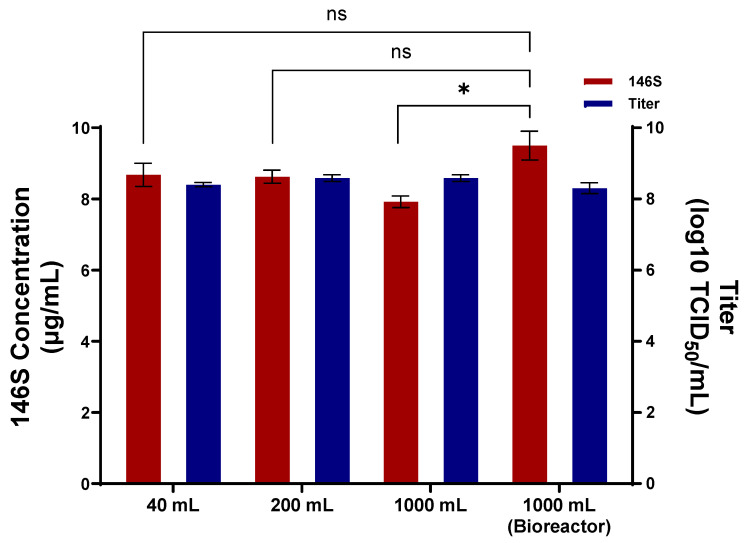
Antigen production efficiency of SAT1 BOT-R during scale-up. Virus titers and 146S antigen yields were compared among flask cultures with working volumes of 40, 200, and 1000 mL and a bioreactor culture operated under the optimized production conditions. Results are shown as mean ± standard deviation. * *p* < 0.05; ns = not significant.

SAT1 BOT-R antigen was produced in flask cultures with working volumes of 40 mL, 200 mL, and 1000 mL. The antigen yields obtained from each flask volume were 8.7 μg/mL, 8.6 μg/mL, and 7.9 μg/mL, respectively (Figure 2).

For bioreactor production, after the suspension cells had settled, 90% of the spent medium was replaced with fresh medium, the pH was adjusted to 8.0, and the culture was subsequently inoculated with SAT1 BOT-R. After harvest, the antigen yield was determined to be 9.5 μg/mL (Figure 2). Antigen production at 40 mL, 200 mL flask, and bioreactor scales was higher than that obtained from the 1000 mL flask.

### 3.3. Inactivation Kinetics of SAT1 BOT-R

**Figure 3:**
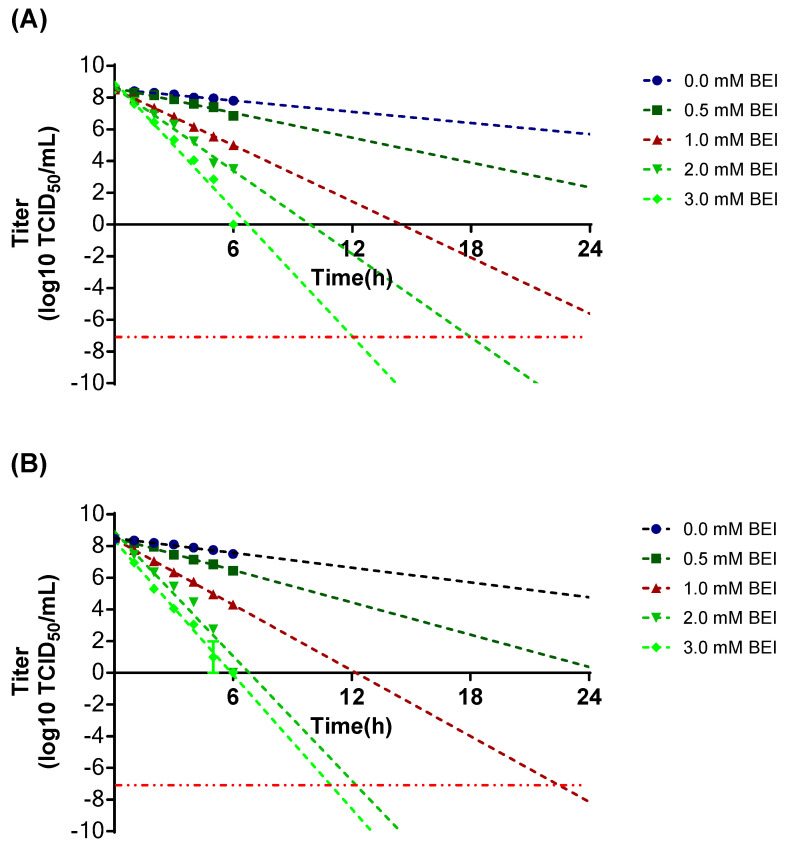
Inactivation kinetics of SAT1 BOT-R under different BEI treatment conditions. Virus-containing supernatants were incubated with the indicated concentrations of binaryethylenimine (BEI), and residual infectivity was monitored over time at 26 °C (**A**) or 37 °C (**B**). Linear regression lines were used to estimate the time required to reach the target inactivation level (−7 log10 TCID_50_/mL), which is indicated by a horizontal dotted line.

The supernatant of SAT1 BOT-R obtained from a bioreactor was used to establish the optimal conditions for SAT1 BOT-R inactivation according to temperature and BEI concentration. At 26 °C, complete inactivation to −7 log10 TCID_50_/mL was achieved within 24 h from 2 mM BEI onward, whereas at 37 °C, complete inactivation to −7 log10 TCID_50_/mL was achieved within 24 h from 1 mM BEI onward (Figure 3).

To determine whether antigen loss occurred during the inactivation process, antigen recovery was assessed after BEI treatment. Under the condition of 2 mM BEI at 26 °C, antigen content decreased by 6%, whereas under the condition of 1 mM BEI at 37 °C, antigen content decreased by 28%. However, even in the untreated control group, antigen content decreased by 25%. These findings indicate that antigen loss was more strongly associated with heat treatment than with BEI treatment itself (Table 1). Therefore, the optimal inactivation condition for SAT 1 BOT-R was determined to be treatment with 2 mM BEI at 26 °C.

### 3.4. Immunogenicity of the SAT1 BOT-R Vaccine Antigen in Pigs

**Figure 4:**
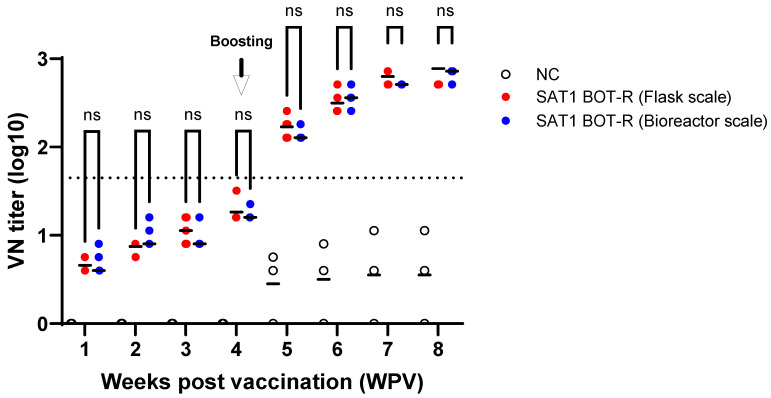
Comparison of antibody responses induced by SAT1 BOT-R antigens produced in flasks and in a bioreactor. Pigs were immunized with experimental vaccines prepared from SAT1 BOT-R antigens generated in the two production systems, and serum samples were analyzed over time. A VN titer of >1.65 log was defined as positive, as indicated by the dotted line. Red and blue circles represent antibody titers induced by antigens produced in shake-flask and bioreactor cultures, respectively. ns, not significant.

The immunogenicity of SAT1 BOT-R vaccine antigen was evaluated in pigs using experimental vaccines prepared either from antigen produced at a 1 L scale in flask culture or from antigen produced in a 1 L bioreactor culture (Figure 4). The experimental pigs were selected as 8-week-old animals seronegative for FMDV. Blood samples were collected once weekly for a total of eight times after vaccination, and a booster vaccination was administered at 4 weeks after the primary immunization. Virus neutralization test results showed no difference between antigens produced at flask scale and those produced in the bioreactor. After two immunizations, neutralizing antibody titers reached a median titer of 1:500, indicating a level considered sufficient for protection. Regardless of the number of vaccinations, no statistically significant difference was observed between the antigens produced at flask scale and those produced in the bioreactor.

## 4. Discussion

Although SAT3 remains restricted to the African continent, SAT1 and SAT2 have recently shown a tendency to spread into the Middle East [18]. In particular, SAT1 outbreaks were reported in Qatar in 2023 [1] and subsequently in Iraq, Bahrain, and Kuwait in 2025 [19]. Following the report of the first SAT1 outbreak in swine in Cyprus in April 2026, the number of outbreaks increased to 85, while 22 outbreaks were reported in Greece. These viruses were identified as belonging to the SAT1/III topotype [20]. In addition, SAT1 outbreaks have also been reported in China this year; however, the exact topotype has not yet been determined [21]. This expanding geographic distribution indicates that the potential introduction of SAT1 into Republic of Korea cannot be excluded. Similar situations have been observed for other transboundary animal diseases, such as African swine fever and lumpy skin disease, which were once considered geographically limited to Africa but were later detected in Republic of Korea in 2019 and 2023, respectively. Therefore, SAT1 should not be regarded as a remote threat, and preemptive preparedness is required to enable rapid response in the event of introduction and thereby protect the livestock industry.

A previous study demonstrated that immunization of pigs with the SAT1 BOT-R antigen conferred protective efficacy against viral challenge [14]. However, for SAT1 BOT-R to be used as a vaccine seed strain for an antigen bank or related applications, it is first necessary to ensure that sufficient amounts of the vaccine antigen, namely intact FMDV particles (146S), can be produced and stably scaled up. In addition, the inactivation kinetics of the virus during antigen production must be carefully evaluated to confirm complete and reliable inactivation. The present study was conducted to address these requirements.

For FMD vaccines, the critical determinant is not the VP1 protein alone, nor individual neutralizing epitopes or pentamers, but rather the efficient production of intact 146S virions, which constitute the relevant vaccine antigen [8,9,10]. Traditionally, FMDV antigen content was quantified by sucrose density gradient ultracentrifugation combined with continuous absorbance monitoring [22,23]. However, when a large number of samples must be analyzed, as in the present study, this conventional method becomes impractical because only a limited number of samples can be processed at a time. Therefore, we employed an HPLC-based method equipped with a gel filtration column to quantify viral particles in an automated manner. This analytical approach has already been adopted by other research groups, including those in Argentina and China [16,24].

As reported previously [25], SAT1 BOT-R antigen was not detected in the absence of calcium in the present study, whereas antigen became detectable when calcium was added simultaneously with the virus to the cells. Although the effect of metal ions on FMDV antigen productivity has not been extensively characterized, calcium has been reported to exert a positive effect on FMDV stability [26]. In addition, calcium supplementation during chromatographic purification has been shown to improve viral stability [27]. In hepatitis A virus, calcium treatment has also been reported to enhance receptor binding and increase viral infectivity [28]. Taken together, these observations suggest that, in SAT1 BOT-R, calcium may increase antigen yield by enhancing the interaction between the virus and cellular receptors. In contrast, another SAT vaccine candidate, SAT2 ZIM-R, showed an antigen yield of approximately 8 μg/mL even in the absence of calcium [29]. The reason SAT1 BOT-R requires calcium whereas SAT2 ZIM-R does not remain unclear and warrants further investigation.

The SAT1 strains currently circulating in Europe belong to topotype III [20]. Since the SAT1 BOT-R strain used in this study also belongs to topotype III, it is expected that this vaccine strain would provide protective efficacy against the currently circulating field viruses.

In the present study, the highest antigen yields were observed at pH 8.0 or pH 8.5, whereas the antigen level declined sharply at pH 9.0. In a previous study, SAT2 ZIM-R maintained its antigen level even at pH 9.0 [29]. Therefore, the lower pH stability of SAT1 BOT-R observed here may be related to its lower intrinsic stability.

The antigen yield of approximately 9.5 μg/mL obtained from virus-containing supernatant in the bioreactor is notably high [20,30,31,32,33]. Previous studies have frequently reported antigen yields in the range of approximately 1–3 μg/mL. Moreover, previous studies have suggested that production scale has only a limited impact on FMDV antigen productivity, with comparable yields achieved across systems ranging from small-scale bioreactors to ton-scale manufacturing platforms [33,34]. Therefore, the approximately 3- to 9-fold higher antigen yield observed in the present study compared with conventional production levels is highly advantageous from the standpoint of manufacturing economy and may substantially improve the feasibility of large-scale vaccine production.

In influenza virus production, viral propagation has in some cases been reported to be more efficient in flask culture than in bioreactor culture [35]. Likewise, in SAT2 ZIM-R, the antigen yield obtained in a bioreactor was lower than that obtained in flask culture [29]. In contrast, in the present study, antigen yields were similar in flask and bioreactor cultures during scale-up. Based on these findings, SAT1 BOT-R appears to be less stable than SAT2 in terms of thermal and pH stability, yet relatively resistant to the physical stress generated by bioreactor agitation.

Evaluation of SAT1 BOT-R inactivation showed that treatment with 2 mM BEI at 26 °C for 24 h resulted in only a 6% loss of antigen, indicating that the antigen is highly stable under these conditions. By contrast, under the 37 °C condition, a 28% reduction in antigen was observed. However, because approximately 25% of this decrease was attributable to the temperature condition itself, these findings suggest that SAT1 BOT-R has poor thermal stability. Accordingly, exposure of SAT1 BOT-R to 37 °C should be minimized during vaccine production in order to achieve consistently high antigen yields.

When the immunogenicity of flask-derived antigen and bioreactor-derived antigen was compared in pigs, no significant difference was observed. This finding indicates that the vaccine efficacy was not affected by the antigen production platform. In other words, the physical stress caused by the bioreactor impeller does not appear to induce substantial antigen damage. In the present study, both antigens induced neutralizing antibody titers reaching a median titer of 1:500 after two vaccinations, indicating that both production methods yielded antigens capable of conferring effective protection against homologous virus, given the correlation between neutralizing antibody titers and protective efficacy [36].

## 5. Conclusions

In conclusion, SAT1 BOT-R is a promising candidate vaccine seed strain for SAT1 antigen banking and future domestic FMD vaccine production. These results provide a practical basis for strengthening national preparedness against the potential introduction of SAT1 FMDV into Republic of Korea.

## Figures and Tables

**Table 1:** Changes in SAT1 BOT-R antigen content after BEI treatment at different temperatures. Residual 146S antigen levels were measured after incubation with the indicated BEI concentrations at 26 °C or 37 °C for the designated times. Values are presented as mean ± standard deviation, and antigen retention was compared with that of untreated controls.

BEI Concentration	26 °C			37 °C		
	0 h	6 h	24 h	0 h	6 h	24 h
0.0 mM BEI	17.0 ± 0.12	17.0 ± 0.11	17.4 ± 1.37	17.0 ± 0.12	17.1 ± 0.51	12.7 ± 0.72
0.5 mM BEI	17.0 ± 0.12	17.2 ± 0.10	16.6 ± 0.30	17.0 ± 0.12	16.9 ± 0.47	12.6 ± 0.47
1.0 mM BEI	17.0 ± 0.12	17.0 ± 0.35	16.0 ± 1.05	17.0 ± 0.12	16.6 ± 0.13	12.3 ± 0.50
2.0 mM BEI	17.0 ± 0.12	16.9 ± 0.52	15.9 ± 0.67	17.0 ± 0.12	16.7 ± 0.77	11.8 ± 0.48
3.0 mM BEI	17.0 ± 0.12	16.7 ± 0.41	15.0 ± 0.62	17.0 ± 0.12	16.4 ± 0.96	11.7 ± 0.88

## Data Availability

The original contributions presented in this study are included in the article. Further inquiries can be directed to the corresponding author.
